# Effect of bevacizumab against cystic components of brain tumors

**DOI:** 10.1002/cam4.2537

**Published:** 2019-09-09

**Authors:** Fumiyuki Yamasaki, Manish Kolakshyapati, Motoki Takano, Ushio Yonezawa, Ikuno Nishibuchi, Nobuki Imano, Akira Taguchi, Shumpei Onishi, Vishwa Jeet Amatya, Yukio Takeshima, Yasushi Nagata, Kaoru Kurisu, Kazuhiko Sugiyama

**Affiliations:** ^1^ Department of Neurosurgery Graduate School of Biomedical and Health Sciences Hiroshima University Hiroshima Japan; ^2^ Department of Radiation Oncology Graduate School of Biomedical and Health Sciences Hiroshima University Hiroshima Japan; ^3^ Department of Pathology Graduate School of Biomedical and Health Sciences Hiroshima University Hiroshima Japan; ^4^ Department of Clinical Oncology & Neuro‐oncology Program Hiroshima University Hospital Hiroshima Japan

**Keywords:** bevacizumab, cyst, glioblastoma, metastatic brain tumor, primary brain tumor

## Abstract

**Background:**

Bevacizumab improves symptoms via reducing the peritumoral edema and/or normalizing blood brain barrier, and occasionally via reducing the tumor size. However, the effect against active cystic components has not been documented yet.

**Materials and Methods:**

Between 2008 and 2018, 139 patients with primary or metastatic brain tumors were treated with bevacizumab (BEV) in our institution. The images and symptoms before and after administration of BEV were examined, and changes in size of cysts were evaluated as follows: CR (complete disappearance), PR (reduction by ≥50%), MR (reduction by ≥25%), SD (size change <25%), PD (increase by ≥25%). The effect of BEV on tumor itself was determined according to Response Assessment in Neuro‐Oncology criteria.

**Results:**

Of the 139 patients, 21 (15.1%) had cystic components. The best responses of cysts to BEV treatment were as follows: CR 6, PR 7, MR 4, SD 4. The group of patients with progressively increasing cysts prior to BEV treatment had significant cyst size reduction compared to stable cyst size groups, at initial imaging after BEV (mean 62.6% vs 22.5%, *P* = .0055) and at best response timing (mean 76.3% vs 32.8%, *P* = .0050). Patients with cysts showed significant improvement in symptoms after the treatment with BEV compared to patients without cysts (*P* = .0033). However, response rate was not different between patients with or without cysts. Overall survival after starting BEV was not different between glioblastoma patients with or without cysts.

**Conclusion:**

Bevacizumab is effective against progressively increasing cysts. Although cysts reduction effect and tumor response and/or overall survival are independent, BEV may be effective in patients who are symptomatic due to cyst enlargement.

## Graphical abstract

Patients with cyst showed significant improvement in symptoms after the treatment with bevacizumab (BEV) compared to patients without cyst. Bevacizumab is effective against progressively increasing cysts. Although cyst reduction effect and tumor response and/or overall survival are independent, BEV may be effective in patients who are symptomatic due to cystic enlargement.

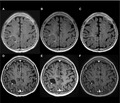

## 1 INTRODUCTION

Bevacizumab (BEV) is a monoclonal antibody that targets vascular endothelial growth factor (VEGF). Bevacizumab binds and inactivates all isoforms of VEGF, and works as an anti‐angiogenic agent.^1^ The results of phase III studies regarding the combination of BEV and cytotoxic drugs against some cancers show improvement in progression‐free survival and/or overall survival in patients with advanced cancers in first‐line and/or second line settings, including metastatic colorectal cancer, advanced non‐squamous non‐small cell lung cancer, metastatic breast cancer, renal cell carcinoma, and epithelial ovarian cancer.^2^

Glioblastoma, the most common malignant primary neoplasm of the central nervous system, also expresses VEGF and its receptors. Vascular endothelial growth factor has been investigated as a potent mediator of brain tumor angiogenesis, vascular permeability, and glioma growth. Bevacizumab was approved for the treatment of recurrent glioblastoma in 2009. However, despite its approval, promising preclinical data and early clinical trials, multiple randomized large phase 3 clinical trials have failed to show survival benefit of BEV in patients with glioblastoma.^3^, ^4^ While, some patient group may have benefit from BEV treatment, it is of high clinical value to predict the patients who will respond to BEV treatment.

Cystic formation is sometimes observed in glioblastoma and other brain tumors.^5^, ^6^ As the intracranial volume is fixed, both tumor itself and cystic components contribute to increase intracranial pressure. Recent reports showed that BEV decreased the cysts' size of vestibular schwannoma, a representative tumor of extra‐axial brain tumor, in neurofibromatosis type 2 patients.^7^, ^8^ However, there has been no report about BEV effect against cysts of intra‐axial brain tumor including high grade glioma. In this study, we focused on the cystic components of intra‐axial brain tumors. We clearly showed that BEV is effective against progressively increasing cysts of intra‐axial tumors.

## 2 MATERIALS AND METHODS

Our institutional review board approved this retrospective study (IRB No. 2953/ E‐1585). Between September 2008 to December 2018, 139 patients with recurrent brain tumors were treated with BEV. Recurrence was defined as imaging progression with or without symptomatic progression. Their age ranged from 1 to 94 years (mean 49.0 years, median 54 years), and 79 were male, 60 were female patients. Histological diagnosis was as follows: 65 glioblastoma (including 1 epithelioid glioblastoma, 1 H3F3A G34R‐mutant glioblastoma), 8 anaplastic astrocytoma, 12 anaplastic oligodendroglioma, 4 diffuse astrocytoma, 18 diffuse midline glioma, 5 pilocytic astrocytoma, 3 anaplastic ependymoma, 5 embryonal tumors (4 medulloblastoma, 1 embryonal tumor with multilayer rosettes [ETMR]), 2 diffuse leptomeningeal glioneuronal tumors (DL‐GNT), 1 germ cell tumors, 16 metastatic tumors (15 lung, 1 unknown). Bevacizumab administration schedule was as follows: 10 mg/kg at every 2 weeks, or 15 mg/kg at every 3 weeks. Treatment schedule of BEV was modified in each patient in the event of development of 3+ proteinuria as an adverse effect.

All patients underwent magnetic resonance imaging (MRI) study which included non‐enhanced T1‐weighted imaging, T2‐weighted imaging, fluid‐attenuated inversion recovery (FLAIR), diffusion‐weighted imaging, and gadolinium‐enhanced T1‐weighted imaging with slice thickness 7 mm or less. In this study, we defined cystic lesions as high intense lesion with long axis of 1 cm or more on T2‐weighted imaging, with thin wall defined by T2 low band and/or gadolinium‐enhanced T1‐weighted imaging. We excluded necrotizing cysts defined as enhancement inside the enhanced cyst wall on gadolinium enhanced T1WI, or obvious heterogeneity inside the cysts on FLAIR imaging. Overall, in our case series 21 patients were defined to have cystic lesions before BEV treatment. The area of cysts was defined as the product of the long axis and the length of the axis perpendicular to it, also called the short axis.

First, we compared the images just before administration of BEV with the images 1‐3 months prior. We defined patients with cysts as follows: active cysts group (cysts size was increase by ≥25%), inactive cysts group (cysts size was increase by <25%). Second, we compared the images just before administration of BEV and images within 2 months after BEV treatment. Third, we compared the images just before administration of BEV and at the points when the size of cysts was smallest (best response point of cysts). Changes in cysts size were evaluated as follows: CR (complete disappearance), PR (reduction by ≥50%), MR (reduction by ≥25%), SD (size change <25%), PD (increase by ≥25%). We also evaluated the effect of BEV on the tumor itself according to (Response Assessment in Neuro‐Oncology) RANO criteria. Best response to BEV was assessed in each patient.

Then, we evaluated the BEV effect against symptoms of patients. Karnofsky performance status (KPS) before and after 2 courses of BEV administration was evaluated. Effect of BEV against symptom was defined as follows: improved (increase KPS by at least 10), no change, worsened (decrease KPS by at least 10). Seven patients were administrated only one course because of progressive disease (1), adverse events (3), patient refusal (2) or switching regimen (1), and evaluated KPS after one course of BEV. We also analyzed the prognosis in recurrent glioblastoma patients after BEV administration.

Statistical analyses were performed with PRISM version 5.0 (GraphPad Software Inc). Cysts increment rate/reduction rate were calculated, and for statistical analysis Mann‐Whitney *U* test and Fisher's exact text were used. Symptom changes between patients with cysts and without cysts were evaluated by Fisher's exact text.

## 3 RESULTS

Among 139 patients, 21 patients (12 males, 9 females; age range 2‐77, mean 40.4, median 44‐years‐old) had cystic components. Characteristics of patients with cysts and without cysts are summarized in Table 1. Karnofsky performance status improvement was observed in 71.4% of patients with cysts, while KPS improvement was observed in 35.6% of patients without cysts, and the difference was statistically significant (*P* = .0033, Fisher exact test). However, response rate of solid part of tumor was almost same between tumor with cysts group and without cysts group (57.1% and 57.6%, respectively).

**Table 1:** Summary of patients with or without cystic components

	Total	Without cysts	With cysts	*P*
Age				>.05
Range	1‐94	1‐94	2‐77	
Median	54	56.5	47	
Gender				>.05
M	79	67	12	
F	60	51	9	
Diagnosis				>.05
Grade IV glioma	83	73	10	
Grade III glioma	23	20	3	
Grade I and II glioma	9	6	3	
Miscellaneous tumors	8	6	2	
Metastatic tumor	16	13	3	
KPS before BEV				>.05
<60	47	40	7	
70	29	23	5	
≥80	65	55	9	
KPS after BEV				.0033
Improved	57	42	15	
No change	66	60	6	
Worsened	16	16	0	
Best response to BEV				>.05
CR	19	18	1	
PR	61	50	11	
SD	40	31	9	
PD	19	19	0	

Abbreviations: BEV, bevacizumab; CR, complete response; exam., examination; KPS, Karnofsky performance status; PD, progressive disease; PR, partial response; SD, stable disease.

Histological diagnosis of tumors with cysts was as follows: 7 glioblastoma, 1 anaplastic astrocytoma, 2 anaplastic oligodendroglioma, 3 diffuse midline glioma, 3 pilocytic astrocytoma, 1 ETMR, 1 DL‐GNT, 3 metastatic tumors. The characteristics of primary or metastatic brain tumor with cysts, symptoms, and response to BEV are summarized in Table 2.

**Table 2:** Summary of brain tumor patients with cysts

Diagnosis	Age	Sex	Cysts size 1‐3 mo before BEV (mm)	Cysts size immediately before BEV (mm)	Pre‐treatment cysts size change	Pre‐treatment solid tumor change	Progression of Symptom before BEV	Cysts size at first exam. after BEV (mm)	Initial response of cysts on BEV	Smallest cysts size during BEV (mm)	Best response of cysts on BEV	Best response of solid tumor on BEV	Pre‐treatment KPS	Post‐treatment KPS	Main symptom	Post‐treatment symptom
Glioblastoma	61	F	19 × 12	25 × 18	PD	PD	Yes	20 × 12	MR	0 × 0	CR	PR	70	80	Hemiparesis	Improved
Glioblastoma	47	F	8 × 8	13 × 10	PD	PD	Yes	0 × 0	CR	0 × 0	CR	PR	60	70	Hemiparesis	Improved
Glioblastoma	49	M	22 × 11	31 × 21	PD	PD	Yes	24 × 16	MR	21 × 14	PR	SD	50	70	Hemiparesis	Improved
Glioblastoma	54	F	32 × 21	34 × 21	SD	SD	Yes	33 × 23	SD	33 × 20	SD	SD	50	50	Hemiparesis	No change
Glioblastoma	56	M	23 × 12	24 × 12	SD	PD	No	23 × 12	SD	23 × 12	SD	CR	90	90	Headache/dysarthria	No change
Glioblastoma	47	M	25 × 12	25 × 12	SD	PD	Yes	22 × 11	SD	22 × 11	SD	PR	70	70	Hemiparesis	No change
Glioblastoma	71	M	7 × 5	10 × 6	PD	SD	Yes	8 × 5	MR	8 × 5	MR	SD	90	90	Dementia	No change
Anaplastic astrocytoma	33	F	0 × 0	26 × 17	PD	PD	Yes	0 × 0	CR	0 × 0	CR	PR	70	90	Hemiparesis	Improved
Anaplastic oligodendroglioma	54	F	6 × 6	11 × 10	PD	PD	No	9 × 6	PR	0 × 0	CR	PR	80	90	Aphasia	Improved
Anaplastic oligodendroglioma	44	F	19 × 18	28 × 23	PD	PD	Yes	21 × 16	MR	21 × 16	MR	SD	70	80	Hemiparesis	Improved
Diffuse midline glioma	12	M	13 × 10	18 × 13	PD	SD	No	3 × 3	PR	0 × 0	CR	SD	80	80	Hemiparesis	No change
Diffuse midline glioma	7	F	12 × 6	19 × 15	PD	PD	Yes	15 × 10	MR	15 × 6	PR	SD	60	90	Headache/vomiting	Improved
Diffuse midline glioma	18	M	12 × 6	19 × 10	PD	SD	Yes	11 × 4	PR	11 × 4	PR	PR	80	90	Headache/hemianopsia	Improved
Pilocytic astrocytoma	11	F	13 × 13	13 × 13	SD	SD	Yes	13 × 13	SD	13 × 13	SD	SD	80	90	Tunnel vision	Improved
Pilocytic astrocytoma	27	M	16 × 11	22 × 15	PD	SD	Yes	10 × 7	PR	7 × 6	PR	PR	80	100	Diplopia/dizziness	Improved
Pilocytic astrocytoma	34	M	50 × 24	56 × 24	SD	SD	Yes	41 × 14	PR	21 × 10	PR	PR	50	70	Tetraparesis/vomiting	Improved
ETMR	2	M	0 × 0	18 × 16	PD	SD	Yes	7 × 6	PR	0 × 0	CR	SD	60	80	Headache/vomiting	Improved
DLGNT	19	M	12 × 12	12 × 12	SD	PD	No	11 × 7	MR	11 × 7	MR	SD	50	70	Headache/vomiting	Improved
Metastatic tumor (SCLC)	58	M	22 × 20	25 × 22	PD	SD	Yes	21 × 21	SD	17 × 17	MR	PR	70	80	Hemiparesis	Improved
Metastatic tumor (NSCLC)	68	F	25 × 20	28 × 23	PD	SD	No	22 × 14	PR	22 × 14	PR	PR	80	80	Headache	No change
Metastatic tumor (NSCLC)	77	M	13 × 12	13 × 12	SD	SD	Yes	11 × 9	MR	10 × 5	PR	PR	80	90	Hemiparesis	Improved

Abbreviations: BEV, bevacizumab; CR, complete response; DLGNT, diffuse leptomeningeal glioneuronal tumor; exam., examination; ETMR, embryonal tumor with multilayer rosettes; KPS, Karnofsky performance status; MR, moderate response; NSCLC, non‐small cell lung cancer; SCLC, small cell lung cancer; PD, progressive disease; PR, partial response; SD, stable disease.

Before administration of BEV, cysts size condition was as follows: PD 14, SD 7. We subdivided patients with cysts as follows: active cysts group (PD), N = 14, inactive cysts group (SD), N = 7. On the first imaging following administration of BEV, the response of cysts was as follows: CR 2, PR 7, MR 7, SD 5. The best response of cysts to BEV treatment were as follows: CR 6, PR 7, MR 4, SD 4. No cyst showed increase in size (PD). We then sub‐divided patients with cysts as responder (MR/PR/CR) and non‐responder (SD), and statistically evaluated the difference between active and inactive cysts group. Responders were more frequently observed in active cysts group compared to inactive cysts group both at initial image after administration of BEV and best response point (*P* = .0251, .0058, respectively, Fisher's exact test).

**Figure 1:**
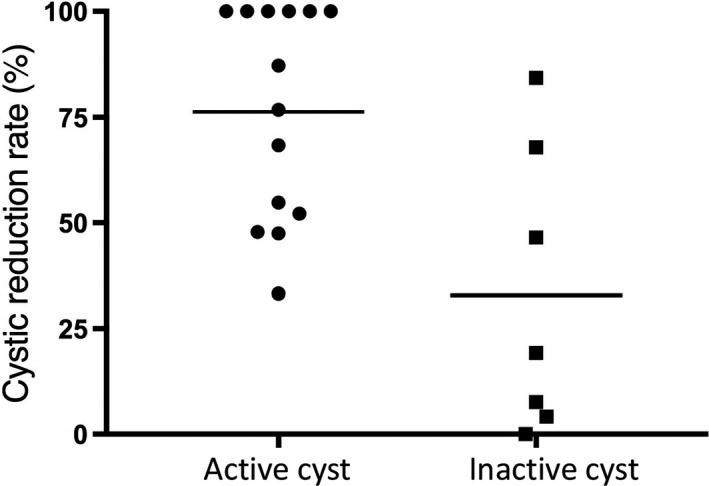
Scatter dot plot of cysts size reduction rate between prior to and best response after bevacizumab treatment. Comparison was performed between active and inactive cysts group, and cysts size reduction rate is much higher in active cysts group (mean 76.3% vs 32.8%, *P* = .0050, Mann‐Whitney *U* test)

We subsequently compared the actual change in cysts size between active and inactive cysts group. The group of patients with active cysts responded to BEV and showed significant cysts size reduction compared to inactive cysts group, at initial imaging after BEV (mean 62.6% vs 22.5%, *P* = .0055) and at best response timing (mean 76.3% vs 32.8%, *P* = .0050; Figure 1). The effect on the solid portion of tumor as per RANO was as follows: CR 1, PR 11, SD 9. We divided patients into 2 groups, solid part responder and non‐responder. The response of solid component was not associated with cysts response (*P* = .3972).

**Figure 2:**
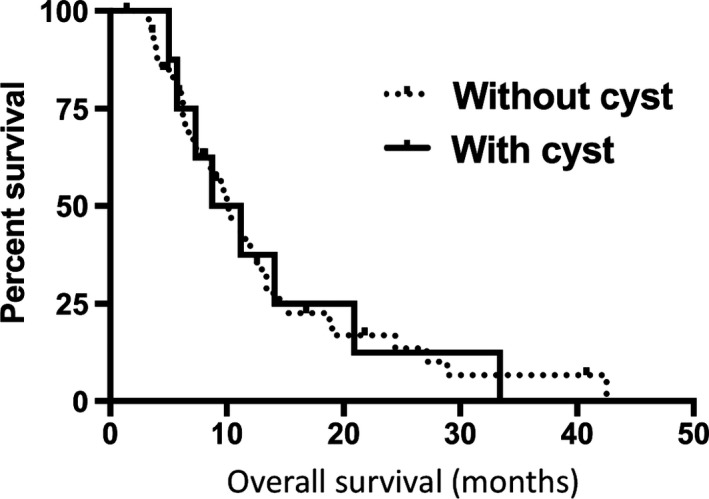
Kaplan‐Meier survival curve of recurrent glioblastoma after starting bevacizumab treatment. There was no statistical difference between tumor with cysts and without cysts (*P* = .9920)

Finally, we compared the prognosis between glioblastoma patients with cysts and glioblastoma patients without cysts. The overall survival was calculated from the initiation date of BEV treatment. Unfortunately, we could not find any statistical difference between both groups (Figure 2).

**Figure 3:**
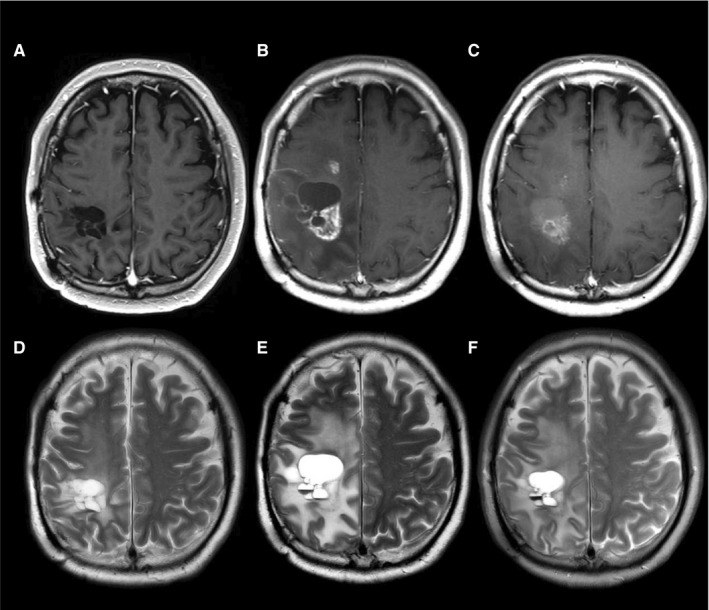
Gadolinium enhanced T1‐weighted imaging (A‐C) and T2‐weighted imaging (D‐F) of 49‐year‐old men with recurrent glioblastoma, isocitrate dehydrogenase‐mutant. Three months before (A/D), immediately before (B/E), 2 wk after (C/F) bevacizumab (BEV) treatment. His Karnofsky performance status worsened from 90% to 50% before BEV treatment resulting from progression of right hemiparesis, and improved to 80% after BEV treatment

**Figure 4:**
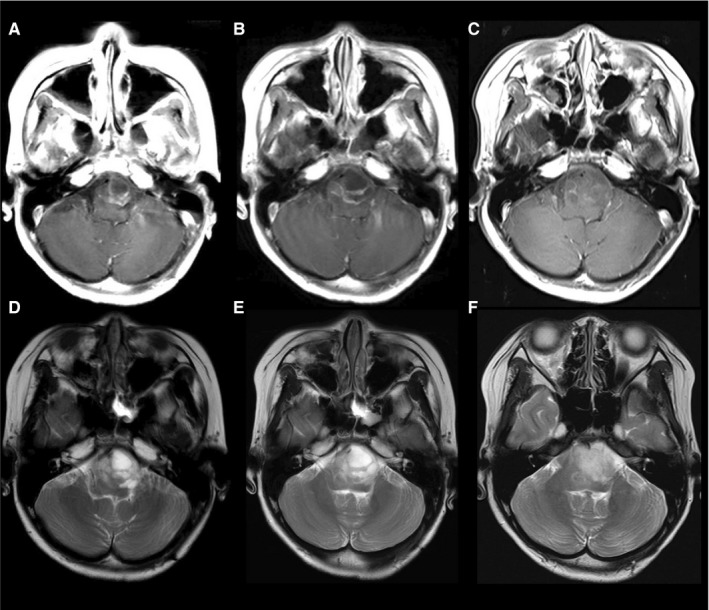
Gadolinium enhanced T1‐weighted imaging (A‐C) and T2‐weighted imaging (D‐F) of 12‐year‐old boy with recurrent diffuse midline glioma, H3 K27M‐mutant. One month before (A/D), immediately before (B/E), 1 mo after (C/F) bevacizumab (BEV) treatment. Cysts size increased before BEV (from A/D to B/E), while decreased after treatment with BEV (from B/E to C/F). However, T2/FLAIR high tumor size increased during both periods (from A/D to B/E, and from B/E to C/F). His dysphagia improved after BEV treatment

**Figure 5:**
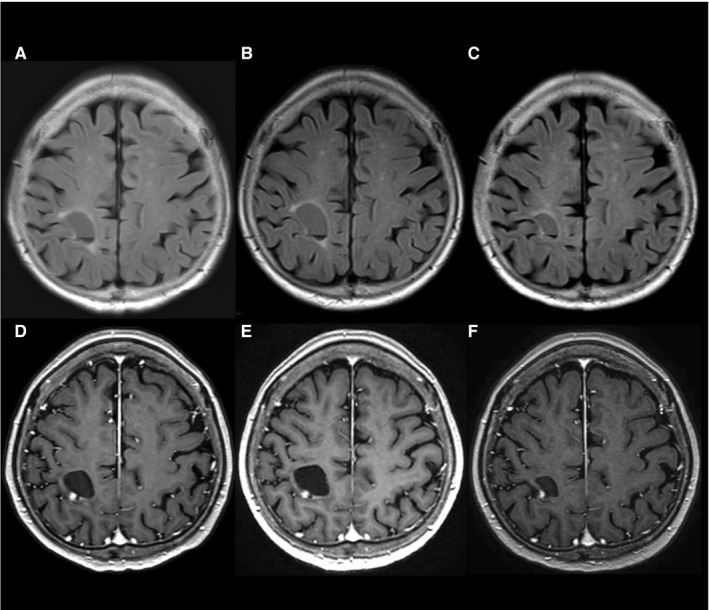
Fluid‐attenuated inversion recovery (A‐C) and gadolinium enhanced T1‐weighted imaging (D‐F) of 68‐year‐old woman with metastatic brain tumor from lung adenocarcinoma. Three months before (A/D), immediately before (B/E), 1 mo after (C/F) bevacizumab (BEV) treatment. Enhanced lesion was not changed during this treatment period. Cysts size increased before BEV (from A/D to B/E), while decreased after treatment with BEV (from B/E to C/F). Her symptom of slight headache improved after BEV treatment

Representative cases are presented in Figures 3, 4, 5.

## 4 DISCUSSION

In this study, we showed that BEV markedly reduces the size of active cystic components of tumor. Because control of intracranial pressure is very important for the treatment of brain tumors, this BEV effect for decreasing cysts size is of high clinical value. Our results showing significant improvement in KPS of tumors with cysts also support the effect of BEV against brain tumor cysts. Therefore, patients with increasing size of tumor cysts could be a good target for BEV treatment.

First‐line use of BEV as standard therapy improved progression‐free survival but did not improve overall survival in patients with newly diagnosed glioblastoma in recent two large randomized phase III trials.^3^, ^4^ Therefore, it is of high clinical value to predict the patients who will benefit from BEV treatment. Two reports showed that calcification after BEV treatment might have significance as a predictor for treatment response.^9^, ^10^ However, calcification itself could be a result of BEV treatment, and this information is not useful at initial stage of treatment but for the late stage of treatment. The information of tumor calcification might be useful for making the decision of continuation with the BEV treatment. In this study, we did not have data about calcification because only a few patients underwent follow‐up computed tomography study. Future studies are necessary to investigate the relationship between tumor calcification and BEV treatment for cystic brain tumors.

In the setting of BEV for recurrent glioblastoma, a randomized phase II trial indicated that BEV combined with lomustine improves survival rate compared with lomustine alone.^11^ Subsequently, a phase III European Organization for Research and Treatment of Cancer trial was conducted to compare lomustine monotherapy with lomustine plus BEV in patients with glioblastoma at first recurrence.^12^ Unfortunately, treatment with lomustine plus BEV did not show survival advantage over treatment with lomustine alone in patients with progressive glioblastoma. Therefore, it is also of high clinical value to predict the patients who will have benefit from BEV treatment. Some reports showed the methods for prediction of BEV effect in recurrent glioblastoma. It is reported that patients with pre‐treatment tumor volume larger than 15 cm^3^, post‐treatment volume larger than 7.5 cm^3^, and percentage change in volume <25% had poorer outcome in patients with recurrent glioblastoma by using post‐contrast enhanced T1‐weight imaging.^13^ However, poorer outcome of larger volume at pre‐treatment is something sensible, and in this study, post‐treatment change however could not be estimated before treatment with BEV. Another report using quantitative T2 relaxation times showed that the degree of change in T2 relaxation time during BEV may be an early response parameter predictive of overall survival.^14^ This method also requires administration of BEV to patients to evaluate the response of tumor to BEV, and could not be a pre‐treatment biomarker. A recent approach using radiomics analyses provided prognostic value for survival and progression in patients with recurrent glioblastoma receiving BEV treatment.^15^ Another report showed the efficacy of histogram analysis of apparent diffusion coefficient obtained from diffusion‐weighted imaging. Pre‐treatment average of apparent diffusion coefficient minimum calculation by histogram analysis is a predictive imaging biomarker for overall survival in patients with recurrent glioblastoma treated with anti‐VEGF monotherapy at first or second relapse.^16^ These methods may give valuable information, however, these methods themselves are complicated and not suitable for generalization.

Our study results did not show any survival advantage of BEV against recurrent glioblastoma with cystic components. Previous report showed that cysts in glioblastoma did not affect prognosis. However, compression by cystic components may limit the prognosis in recurrent glioblastoma, and BEV potentially improved the prognosis to the same level as glioblastoma without cysts. Future studies are necessary to confirm the effect of cysts on prognosis of glioblastoma.

There were some reports about cystic contents of brain tumor and VEGF.^5^, ^6^, ^17^, ^18^ Cysts of metastatic and primary brain tumors including glioblastoma, protoplasmatic astrocytoma, pilocytic astrocytoma, ependymoma, meningioma, and craniopharyngiomas expressed high level of VEGF, and that was not reflected on serum level of VEGF. It was speculated that VEGF may be biologically relevant for the formation of tumor cysts in brain tumors. Bevacizumab might be able to reduce the VEGF inside the cysts, that would result in the decreasing size of the cysts. In our study, 71% of “patients with cysts” showed improvement of KPS after BEV treatment, while only 36% of “patients without cysts” showed improvement of KPS after BEV treatment. Cystic tumors may have more VEGF expression, inactivation of which may be associated with improvement of KPS after BEV treatment. Another report regarding BEV effect against “intra‐axial cystic lesions” included treatment for leukoencephalopathy with calcifications and cysts (LCC).^19^ Although the mechanism is unclear BEV reduces the size of cysts in the patients with LCC. Patients with Coats disease (Coats plus syndrome), a distinct genetic entity from LCC, developed macular edema and intraretinal cysts, and intravitreal BEV could diminish the intraretinal cysts and improve macular edema.^20^, ^21^ Moreover, intravitreal BEV is also effective against cystoid macular edema via other etiology.^22^ These effects against cysts is consistent with our results.

We consider that BEV effects against pleural effusion and ascites may be a common phenomenon preventing local fluid accumulation and/or decreasing effusion. Marked elevation of VEGF was also reported in both malignant pleural effusion and malignant ascites, and considered to be a key molecule.^23^, ^24^ Both malignant pleural effusion and malignant ascites were poor prognostic factor and resulted in decline of quality of life (QOL).^25^, ^26^, ^27^, ^28^ Bevacizumab combined with chemotherapy were reported to be a superior option for patients with both malignant pleural effusion and malignant ascites, and could improve patients' QOL.^28^, ^29^, ^30^ Although VEGF level reportedly was high in malignant pleural effusion, the level of serum VEGF level was not associated with high level of pleural VEGF,^31^ that was consistent with the relationship between VEGF level of brain tumor cysts and serum. While, the serum VEGF levels and ascites VEGF levels were highly correlated in malignant ascites patients,^29^ which may be the result of much higher liquid volume in ascites than pleural effusion and brain tumor cysts.

We acknowledge that our study has limitations. We have only a small number of cases in each tumor type, and the timing of cysts development and previous treatment including chemotherapy and radiotherapy was diverse. We did not have information about VEGF concentration of both serum and cystic components. Volumetric analyses may be the better methods for evaluating cysts size. Progression of symptom could be caused by solid part progression, cystic part progression, or both, and we could not evaluate the etiology of symptom progression accurately. Despite these drawbacks, our results about the effects of BEV against active cystic components is valuable in determining the appropriate patient group for BEV therapy.

## 5 CONCLUSIONS

Bevacizumab is effective against progressively increasing cystic components of primary and metastatic brain tumors and in improving KPS. Although cysts reduction effect and tumor response are independent, BEV administration should be considered for patients who are symptomatic due to cystic enlargement.

Two concise sentences that state the significant conclusion(s) or message of the manuscript;

1. Bevacizumab is effective against progressively increasing cystic component of primary and metastatic brain tumors.
2. The response of solid component after treatment with bevacizumab was not associated with cyst response.

## CONFLICT OF INTEREST

None declared.

## AUTHOR CONTRIBUTIONS

Writing—original draft: FY; writing—review and editing; FY, MK, MT, UY, IN, NI, AT, SO, VJA. YT, YN, KK, KS; conceptualization: FY, MT; data curation: FY, MK, MT, UY, IN, NI, AT, SO, VJA; formal analysis: FY; supervision: YT, YN, KK.
